# Higher body mass index is associated with larger postoperative improvement in patient-reported outcomes following total knee arthroplasty

**DOI:** 10.1186/s12891-021-04512-1

**Published:** 2021-07-24

**Authors:** K Giesinger, JM Giesinger, DF Hamilton, J Rechsteiner, A Ladurner

**Affiliations:** 1 Department of Orthopaedics and Traumatology, Kantonsspital St. Gallen, Rorschacherstrasse 95, 9007 St. Gallen, Switzerland; 2 Innsbruck Institute of Patient-Centered Outcome Research (IIPCOR), Innsbruck, Austria; 3 School of Health and Social Care, Edinburgh Napier Univ, ersity, Edinburgh, Scotland

**Keywords:** Total knee arthroplasty, Obesity, Patient-reported outcome, EQ-5D, WOMAC Score

## Abstract

**Background:**

Total knee arthroplasty is known to successfully alleviate pain and improve function in endstage knee osteoarthritis. However, there is some controversy with regard to the influence of obesity on clinical benefits after TKA. The aim of this study was to investigate the impact of body mass index (BMI) on improvement in pain, function and general health status following total knee arthroplasty (TKA).

**Methods:**

A single-centre retrospective analysis of primary TKAs performed between 2006 and 2016 was performed. Data were collected preoperatively and 12-month postoperatively using WOMAC score and EQ-5D. Longitudinal score change was compared across the BMI categories identified by the World Health Organization.

**Results:**

Data from 1565 patients [mean age 69.1, 62.2% women] were accessed. Weight distribution was: 21.2% BMI < 25.0 kg/m^2^, 36.9% BMI 25.0–29.9 kg/m^2^, 27.0% BMI 30.0–34.9 kg/m^2^, 10.2% BMI 35.0–39.9 kg/m^2^, and 4.6% BMI ≥ 40.0 kg/m^2^. All outcome measures improved between preoperative and 12-month follow-up (*p* < 0.001). In pairwise comparisons against normal weight patients, patients with class I-II obesity showed larger improvement on the WOMAC function and total score. For WOMAC pain improvements were larger for all three obesity classes.

**Conclusions:**

Post-operative improvement in joint-specific outcomes was larger in obese patients compared to normal weight patients. These findings suggest that obese patients may have the greatest benefits from TKA with regard to function and pain relief one year post-op. Well balanced treatment decisions should fully account for both: Higher benefits in terms of pain relief and function as well as increased potential risks and complications.

Trial registration

This trial has been registered with the ethics committee of Eastern Switzerland (EKOS; Project-ID: EKOS 2020–00,879)

## Background

Osteoarthritis (OA) is a major global cause of disability, with the knee being the most frequently affected joint [1]. Total knee arthroplasty (TKA) is successful in alleviating the pain and disability associated with knee OA, and is one of the most commonly performed elective surgical procedures in orthopaedics [2]. The improvements in patient health status achieved by TKA have been reported comparable to coronary revascularisation and renal transplant procedures [3]. Due to its well documented success, the number of TKAs performed increases every year and operative rates are reported to have doubled in the last decade [4, 5].

There is an established association between the patients’ body mass index (BMI) and knee OA, possibly due to increased mechanical loading at the joint [6–8]. Increased BMI is suggested to be one of the main modifiable risk factors of knee OA, given that every kilogram of weight loss leads to a fourfold reduction in the load exerted on the knee with daily activities [7]. Preoperative weight loss was found to have considerable implications for patient burden and cost reduction. [9]. High BMI is also associated with a variety of metabolic disturbances such as coronary heart disease, hypertension and diabetes [7, 10] that could result in systemic risk factors for OA. Consequently, patients with increased BMI are more likely to require total knee arthroplasty (TKA) at younger age than patients with normal BMI [11]. Worldwide levels of obesity (BMI > 30 kg/m^2^ by WHO definition) are rising rapidly [12, 13]. A quarter of the population of developed countries are reported as being obese [14]. By 2030, the respective number of overweight (BMI > 25 kg/m^2^) and obese (BMI > 30 kg/m^2^) adults is projected to be 1.35 billion and 573 million individuals [12, 15]. Although increased BMI has been linked to the need for TKA, the impact of BMI on outcomes after TKA is less well established [2, 6, 8, 16–20]. Patients with an increased BMI have been reported higher in-hospital discharge rates [21] and are accepted to have higher risks of postoperative wound dehiscence [22], infection [16, 22, 23], complications, and revision [6, 20, 24, 25]. These risks are often compounded by the associated comorbidities [20] or other factors like socioeconomic status [26] frequently found in such patients. It is not clear though whether BMI is associated with worse patient reported outcomes following TKA, as both equivalent outcomes [27, 28] and lower absolute post-operative scores [17, 19, 29] have been proposed scaled by BMI class using various metrics.

The objective of this study was to investigate the impact of preoperative BMI on postoperative improvement in pain, function and general health status following total knee arthroplasty (TKA) from the patients’ perspective.

## Methods

The study was performed at a large teaching hospital in Switzerland; a higher-income country with a largely white European population. Ethics approval was obtained from the local ethics committee (ethics committee of Eastern Switzerland EKOS; Project-ID: EKOS 2020–00,879). Data from the local knee arthroplasty register was accessed and all patients undergoing elective primary total knee arthroplasty between 2006 and 2016 were considered for inclusion. Simultaneous bilateral surgery or incomplete data sets were criteria for patient exclusion. No patients were precluded from surgery or restricted from study inclusion due to their BMI. On an individual basis, depending on symptom burden and mobility, morbidly obese patients were referred to weight loss programs prior to surgery. Surgery was performed by consultant orthopaedic surgeons and their supervised trainees. Surgery was performed in spinal or general anesthesia. Antibiotic prophylaxis using 3^rd^ generation cephalosporin was administered in every case. A tourniquet was applied and inflated for insertion of the cemented implant. A medial parapatellar approach was routinely used, with a lateral parapatellar approach and osteotomy of the tibial tubercle in cases with severe valgus deformity. Cemented primary implants (LCS complete or Attune, DePuy-Synthes) were implanted in all cases. A tibia first, ligament balancing technique was employed, with and without computer-navigation (Brainlab, Munich). The cruciate ligaments were routinely sacrificed. No special considerations were made for patients based on their BMI during the routine postoperative standard rehab protocol, which was followed in every case. This allowed unrestricted weight bearing as tolerated on crutches and physiotherapy to improve range of motion and muscle activation without further limitations from day one. VTE prophylaxis was performed for 6 weeks following surgery. Outpatient follow-up visits were routinely performed 2 and 12 months postoperatively, and in 5-year intervals thereafter. Preoperative and 12-month postoperative data were retrieved. Patient reported outcome data were prospectively collected at the time of treatment. The Western Ontario and McMaster Universities Osteoarthritis Index (WOMAC score) was employed for measuring pain, stiffness and function in the respective subscales, the EQ-5D for measuring general health status. The WOMAC score is a widely used three-dimension self-administered patient-reported outcome instrument consisting of 24 questions that are linearly transformed to a 0–100 scale with higher scores indicating more severe impairment. The score has been extensively tested for validity, reliability, feasibility and responsiveness [30, 31]. The EQ-5D [32] is a generic self-report questionnaire. It consists of five questions measuring the patient’s health status and covers self-care, mobility, depression/anxiety, pain and usual activities. A health utility can be calculated from the five questions, with a score of 1 reflecting full health, 0 indicating a health state equaling death and negative values describing health states that patients consider worse than being dead. This widely used questionnaire has shown satisfying measurement characteristics in knee patients [33].

### Statistical analysis

Sample characteristics are given as means, standard deviations or 95% confidence intervals, and ranges. Score change following surgery was compared across the BMI categories identified by the World Health Organization (WHO) [13]: normal weight (BMI < 25.00), overweight (BMI 25.00 to 29.99), class I obesity (BMI 30.00 to 34.99), class II obesity (BMI 35.00 to 39.99), and class III obesity (BMI ≥ 40.00).

To investigate the impact of BMI on postoperative improvement we used linear mixed models with the outcome parameters (WOMAC subscales, EQ-5D) as dependent variables, and the following independent variables: BMI group, time point (preoperative and 12-month follow-up), and the two-way interaction of group-by-time. In such a model, the interaction term indicates a difference in postoperative improvement between BMI groups. We ran pairwise post-hoc tests comparing postoperative improvement between the “normal weight” category and the four other BMI categories. The models also included a first-order autoregressive covariance matrix to account for correlations between repeated measurements. Results are presented as estimated marginal means with their 95% confidence intervals and p-values for the group effect, time effect, and the group-by-time interaction. P-values below 0.05 were considered to be statistically significant. All analyses were conducted in SPSS 24.0.

## Results

### Sample characteristics

**Fig. 1:**
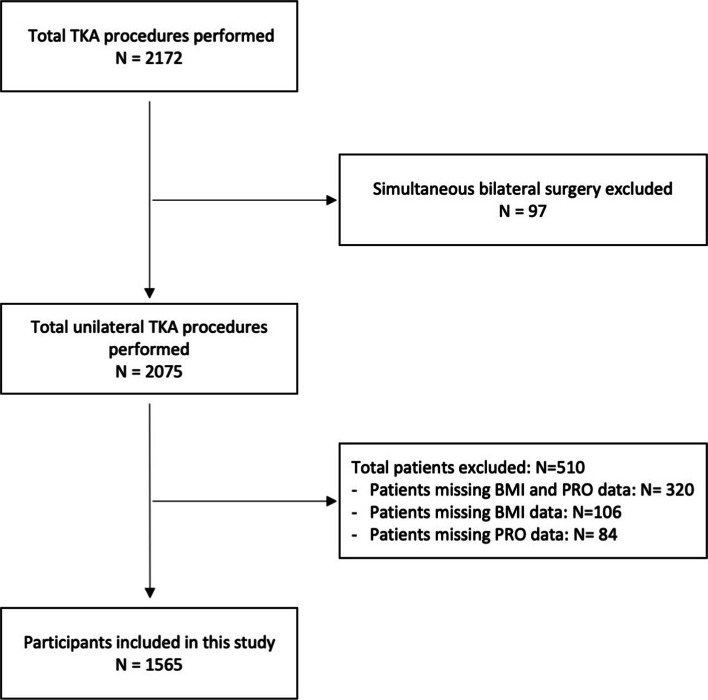
Flowchart on study inclusion

Between February 2006 and December 2016, a total of 2172 patients underwent primary TKA at our institution and were included in the local knee arthroplasty registry. 1565 patients were available for analysis (Fig. 1). There were no statistically significant differences between patients having or missing BMI or PROMs data, indicating that these missing values were random (data not shown).

In this sample, mean patient age was 69.1 years (range 24.8—92.3 years). 62.2% of patients were female, and the right side was affected in 817 cases (52.2%). Response rates for the WOMAC score and the EQ-5D at 12-month were 83.8% and 84.5%, respectively. We found 21.2% of patients (*N* = 332) to be normal weight (BMI < 25.0 kg/m^2^), 36.9% (*N* = 578) were overweight (BMI 25.0–29.9 kg/m^2^), 27.0% (*N* = 423) had class I obesity (BMI 30.0–34.9 kg/m^2^), 10.2% (*N* = 160) had class II obesity (BMI 35.0–39.9 kg/m^2^), and 4.6% (*N* = 72) had class III obesity (BMI ≥ 40.0 kg/m^2^) (Table 1).

**Table 1:** Sociodemographic and clinical patient characteristics at pre-surgery (*N* = 1565)

		**Mean (SD)**	
**Age**		69.1 (10.1)	
		**N**	**(%)**
**Sex**			
Women		974	(62.2%)
Men		591	(37.8%)
**Education**			
Compulsory school		479	(31.2%)
More than compulsory school (A-levels, apprenticeship, university)		1058	(68.8%)
Missing		28	
**Employment status**			
Full-time or part-time		293	18.9%
Retired		730	47.1%
Homemaker		448	28.9%
Other		51	5.1%
Missing		16	
**Smoking**			
No		1277	(82.6%)
Yes		269	(17.4%)
Missing		19	
**Side of implant**			
Left		748	(47.8%)
Right		817	(52.2%)
**BMI (%)**			
≤ 24.99	normal weight	332	(21.2%)
25.00–29.99	pre-obesity	578	(36.9%)
30.00–34.99	class I obesity	423	(27.0%)
35.00–39.99	class II obesity	160	(10.2%)
≥ 40.00	class III obesity	72	(4.6%)
**Computer navigation**			
Yes		931	61.0%
No^a^		595	39.0%
Missing		39	

^a^including *N* = 33 aborted navigations

BMI was associated with sex (*p* < 0.001), with a similar proportion of normal weight patients for both sexes (men 20.5% vs women 21.7%), a higher proportion of pre-obese men (men 43.3% vs women 33.1%), and more women in class I-III obesity (class I: 27.9% women vs 25.5% men, class II 12.2% women vs 6.9% men, class III: 5.1% women vs 3.7% men).

There was a statistically significant association of BMI class and age at the time of surgery (*p* < 0.001). Mean age was 70.5 years in normal weight patients and decreased monotonously to 65.3 years in class III obesity patients.

### WOMAC pain

**Fig. 2:**
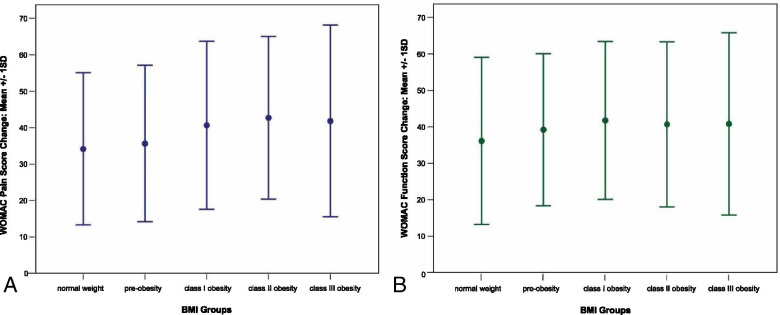
WOMAC pain (**a**) and function (**b**) score change between pre-surgery and 12-month follow-up across BMI groups

WOMAC pain scores differed significantly between BMI groups (*p* < 0.001) with better scores observed in lower BMI categories. Pain scores improved between preoperative assessments and 12-month follow-up (*p* < 0.001). Postoperative improvement was associated with BMI group (*p* < 0.001). In pairwise comparisons of BMI groups, we found improvements in class I, II and III obesity patients (-40.4, -44.2, and -42.3 points) to be statistically significantly (all *p* < 0.05) larger than in normal weight patients (-34.5 points). For further details please see Table 2 and Fig. 2a. All differences remained statistically significant when adjusting for sex and age.

**Table 2:** WOMAC pain, function and total score for different BMI groups (pre-surgery: all scales *N* = 1565; 12-month: pain *N* = 1311, function *N* = 1308, total *N* = 1308)

	Pre-surgery	12 months	Improvement
**BMI**	**Mean (95%CI)**	**Mean (95%CI)**	**Mean (95%CI)**
**WOMAC pain**			
normal: < 25	45.2 (38.7–51.7)	10.7 (2.9–18.5)	34.5 (31.9–37.1)
pre-obesity: 25–30	45.9 (39.3–52.6)	9.8 (3.7–16.0)	36.1 (34.1–38.1)
class I obesity: 30–35	50.5 (43.7–57.2)	10.1 (3.6–16.6)	40.4 (38.0–42.7)*
class II obesity: 35–40	54.3 (41.8–66.8)	10.2 (0.0–22.9)	44.2 (40.3–48.0)*
class III obesity: ≥ 40	54.9 (39.1–70.7)	12.6 (0.0–29.2)	42.3 (36.7–48.0)*
**Group**	**Time**	**Group-by-time interaction**	
*F* = 6.1; *p* < 0.001	*F* = 2458.4; *p* < 0.001	*F* = 6.7; *p* < 0.001	
**WOMAC function**			
normal: < 25	52.6 (50.6–54.6)	16.2 (14.0–18.4)	36.4 (33.8–39.1)
pre-obesity: 25–30	54.5 (53.0–56.0)	15.5 (13.9–17.1)	39.0 (37.0–41.0)
class I obesity: 30–35	57.7 (56.0–59.5)	16.2 (14.3–18.1)	41.6 (39.2–43.9)*
class II obesity: 35–40	61.1 (58.2–63.9)	18.7 (15.6–21.9)	42.3 (38.5–46.2)*
class III obesity: ≥ 40	62.8 (58.6–67.1)	22.4 (17.8–27.0)	40.4 (34.8–46.1)
**Group**	**Time**	**Group-by-time interaction**	
*F* = 8.0; *p* < 0.001	F = 2497.6; *p* < 0.001	*F* = 2.7; *p* = 0.030	
**WOMAC total**			
normal: < 25	50.9 (49.0–52.8)	15.1 (13.1–17.2)	35.8 (33.3–38.3)
pre-obesity: 25–30	52.6 (51.1–54.0)	14.5 (13.0–16.0)	38.1 (36.2–39.9)
class I obesity: 30–35	56.0 (54.3–57.6)	14.9 (13.1–16.7)	41.0 (38.8–43.2)^a^
class II obesity: 35–40	59.1 (56.4–61.8)	16.7 (13.7–19.6)	42.4 (38.8–46.1)^a^
class III obesity: ≥ 40	60.8 (56.8–64.8)	20.2 (15.8–24.5)	40.6 (35.3–46.0)
**Group**	**Time**	**Group-by-time interaction**	
*F* = 7.4; *p* < 0.001	*F* = 2739.4; *p* < 0.001	*F* = 3.6; *p* = 0.006	

^a^ statistically significant (*p* < 0.05) difference in change compared to “normal” weight patients

### WOMAC function

WOMAC function scores differed significantly between BMI groups (*p* < 0.001), with lower BMI groups showing better function. Function significantly improved from pre-surgery to the 12-month follow-up (*p* < 0.001). Postoperative improvement was also associated with BMI group (*p* = 0.030). The pairwise comparisons of BMI groups, showed improvements in class I-II obesity patients (-41.6 and -42.3 points) to be statistically significantly (both *p* < 0.05) larger than in normal weight patients (-36.4 points). Further details are given in Table 2 and Fig. 2b. In sex-and age-adjusted analysis all differences remained statistically significant.

### WOMAC total

For the WOMAC total score we found statistically significant differences between BMI groups overall (*p* < 0.001) with patients with lower BMI reporting better scores, and a general improvement between the preoperative time point and 12-month follow-up (*p* < 0.001).

For the WOMAC total score, postoperative improvement was associated with BMI group (*p* = 0.006). The pairwise comparisons of BMI groups showed statistically significantly (all *p* < 0.05) larger improvements in class I-II obesity patients (-41.0 and -42.4 points) compared to normal weight patients (-35.8 points). Details are given in Table 2. In sex-and age-adjusted analysis all differences remained statistically significant.

### EQ-5D

The EQ-5D utility scores differed between BMI groups (*p* < 0.001), and improved in general from preoperatively to 12-month follow-up (*p* < 0.001). No statistically significant difference between BMI groups was observed regarding postoperative improvement (*p* = 0.066). Adjustment for sex and age resulted in the same statistical differences (Table 3).

**Table 3:** EQ-5D utility values for different BMI groups (pre-surgery *N* = 1521, 12-month *N* = 1286)

EQ-5D utility	Pre-surgery	12 months	Improvement
**BMI**	**Mean (95%CI)**	**Mean (95%CI)**	**Mean (95%CI)**
normal: < 25	0.66 (0.49–0.83)	0.90 (0.31–1.00)	0.24 (0.21–0.27)
pre-obesity: 25–30	0.66 (0.55–0.77)	0.90 (0.79–1.00)	0.24 (0.22–0.26)
class I obesity: 30–35	0.61 (0.45–0.77)	0.89 (0.71–1.00)	0.28 (0.25–0.31)
class II obesity: 35–40	0.58 (0.11–1.00)	0.86 (0.12–1.00)	0.28 (0.23–0.33)
class III obesity: ≥ 40	0.54 (-0.09–1.00)	0.85 (-0.09–1.00)	0.30 (0.24–0.37)
**Group**	**Time**	**Group-by-time interaction**	
*F* = 9.1; *p* < 0.001	*F* = 785.9; *p* < 0.001	*F* = 2.2; *p* = 0.066	

No statistically significant (*p* < 0.05) differences in change between BMI groups

## Discussion

This study highlights that the perceived benefit in terms of joint-specific patient-reported outcome following total knee arthroplasty is larger in obese patients compared to normal weight patients when analyzing post-operative improvement at 12-month follow-up. This association was found irrespective of a possible impact of sex and age.

The literature on the impact of BMI on pain and functional outcomes following TKA is somewhat conflicted. Multiple studies have shown that outcomes after TKA are worse in obese patients than in non-obese patients [10, 18, 34]. Amin et al. [34] reported inferior cross-sectional clinical outcome scores (Knee Society Score) and higher complication rates in morbidly obese patients at a mean follow-up of 38.5 months. They reported a significantly higher rate of radiolucent lines around the implants (notably around the tibial component) and inferior five-year implant survivorship for patients with BMI > 40 kg/m^2^ compared to patients with BMI < 30 kg/m^2^ in their prospective matched pair study. Lash et al. [35] found that patients with BMI > 35 kg/m^2^ had worse preoperative and post-operative functional scores (WOMAC, Oxford Knee Score, High-activity Arthroplasty Score) than patients with BMI < 30 kg/m^2^, but their benefit from surgery measured by the change in functional scores showed no difference. Similarly, Baker et al. [19] reported no differences in OKS improvement among patients with class I, II and III obesity.

In line with our results, Chen et al. [17] suggest greater improvement in the more obese patient groups, with the mean improvement in Oxford Knee Score (OKS) and Knee Society Knee Score (KSKS) at two years follow-up being significantly higher in the morbidly obese group than in the normal group. Greater obesity level was associated with more pain at baseline but greater postoperative pain relief in a study conducted by Li et al. [27]. In this study, the postoperative gain in Physical Component Summary (SF-36) did not differ by BMI level.

Similarly, a recent systematic review by Boyce et al. [6] found that all patients regardless of BMI experienced comparable improvements in knee function following TKA.

The more obese patients in our cohort generally reported a superior improvement in outcome scores. The context of this improvement is that they started with notably poorer preoperative scores. As such although the delta (improvement) was larger, the absolute post-operative score was not superior in the obese patients. When evaluating the clinical benefits of TKA it is crucial to focus on change rather than interpret post-operative data with cross-sectional analysis, as the latter may to a large degree simply reflect pre-operative differences between patients. Change in symptomatology is what is most noticeable to the patient and is the yardstick by which they interpret the benefit of surgery and patients with higher pre-operative symptom burden may have simply more to gain by surgery.

It is important to note that this study did not take into account the increased complication rate in obese patients associated with the intervention. Higher perioperative and postoperative complication risks are well accepted to be greater in obese patients [20, 25, 34, 36]. Complication rates climb further as BMI levels rise into morbidly and super-obese categories [6, 10, 37]. Increased risks include wound related problems like delayed wound healing, superficial wound infection and deep prosthetic joint infection [6, 22, 25, 34, 36, 38]. In addition, obese patients show higher rates of revision, aseptic loosening and radiographic signs of early loosening [6, 34, 38, 39] and more frequent osteolysis or wear [39]. Alongside reduced longevity of the implant, obesity is also associated with reduced life expectancy [40–42]. Patients with BMIs over 35 can expect a significantly reduced life expectancy compared to normal weight individuals. Large scale international study data suggests around 3–4 years are lost to individuals with class I obesity and 10 or more years to those in class III [43–45]. If hesitation to perform surgery in obese patients is due to concerns as to reduced implant longevity, this worry may be partially mitigated by a generally reduced life expectancy. In terms of health economy, even in “high risk” patients, TKA remains a cost-effective intervention [46].

From a clinical perspective, joint replacement surgery is an intervention for end-stage disease. Our findings also support a developmental relationship between OA and obesity, as highly obese patients underwent TKA at a younger age than the normal weight or pre-obese patient population. These considerations are in accordance with the findings of Changulani et al. [47] who stated that for both hip and knee replacement, the age at surgery fell significantly for patients with a BMI > 35 kg/m^2^. It is also likely that the management of increased complications in this population predispose obese patients rather toward a delay of arthroplasty, and it is likely they present even younger to orthopedics than the age at time of surgery suggests.

Strengths of this study include a large sample size which allowed for stratification and comparison of BMI subgroups as suggested by the WHO, the availability of data from a joint-specific questionnaire as well as a general health outcome measure which allowed to demonstrate the differential impact of BMI on outcome after TKA. A limitation is that we had a 28% loss to follow-up rate, 12.6% of patients had to be excluded due to missing PROM questionnaires and 10.9% of patients due to missing BMI values (Fig. 1). However, as collection of BMI values and PROM questionnaires in our series is largely unrelated to BMI category it is unlikely that this may have introduced relevant bias in this study. Another limitation is that the patients’ BMI was only recorded preoperatively and possible weight changes that may have taken place by the 12-month postoperative review were not accounted for. This is potentially important in the light that patients who underwent TKA and lost weight thereafter were reported to have better clinical outcome scores than patients who gained weight in the postoperative period [48]. However, recent literature indicates the patient’s bodyweight to be maintained after TKA [49–51], suggesting a minor possible impact of weight change on our results. The percentage of patients in obesity class II and III was relatively low, compared to reports in other countries with two-thirds of our patient cohort clustered in the overweight / pre-obese and class I obese categories. This however accurately reflects our wider country-specific weight distribution.

## Conclusions

This study demonstrates larger post-operative improvement in joint-specific outcomes in obese patients compared to normal weight patients. Our findings suggest that obese patients may have the greatest benefits from TKA regarding increase of functional capacity and pain relief at one year follow-up. Well balanced treatment decisions in shared decision making should fully account for both: Higher benefits in terms of pain relief and function as well as increased potential risks and complications after TKA.

## Data Availability

The datasets generated and analysed during the current study are not publicly available as access to the relevant institutional database is restricted to the public, but data are available from the corresponding author on reasonable request.

## Abbreviations

OA: Osteoarthritis
TKA: Total knee arthroplasty
BMI: Body Mass Index
WHO: World Health Organisation
WOMAC: Western Ontario and McMaster Universities Osteoarthritis Index
EQ-5D: EuroQol 5D
CR: Cruciate retaining
PRO: Patient reported outcome
